# Newborn Genetic Screening for Hearing Impairment: A Preliminary Study at a Tertiary Center

**DOI:** 10.1371/journal.pone.0022314

**Published:** 2011-07-19

**Authors:** Chen-Chi Wu, Chia-Cheng Hung, Shin-Yu Lin, Wu-Shiun Hsieh, Po-Nien Tsao, Chien-Nan Lee, Yi-Ning Su, Chuan-Jen Hsu

**Affiliations:** 1 Department of Otolaryngology, National Taiwan University Hospital, Taipei, Taiwan; 2 Department of Medical Genetics, National Taiwan University Hospital, Taipei, Taiwan; 3 Department of Pediatrics, National Taiwan University Hospital, Taipei, Taiwan; 4 Department of Obstetrics and Gynecology, National Taiwan University Hospital, Taipei, Taiwan; 5 Graduate Institute of Clinical Genomics, National Taiwan University College of Medicine, Taipei, Taiwan; Charité Universitätsmedizin Berlin, NeuroCure Clinical Research Center, Germany

## Abstract

Universal newborn hearing screening (UNHS) is of paramount importance for early identification and management of hearing impairment in children. However, infants with slight/mild, progressive, or late-onset hearing impairment might be missed in conventional UNHS. To investigate whether genetic screening for common deafness-associated mutations could assist in identifying these infants, 1017 consecutive newborns in a tertiary hospital were subjected to both newborn hearing screening using a two-step distortion-product otoacoustic emissions (DPOAE) screening and newborn genetic screening (NGS) for deafness. The NGS targeted 4 deafness-associated mutations commonly found in the Taiwanese population, including p.V37I (c.109G>A) and c.235delC of the *GJB2* gene, c.919-2A>G of the *SLC26A4* gene, and mitochondrial m.1555A>G of the 12S rRNA gene. The results of the NGS were then correlated to the results of the NHS. Of the 1017 newborns, 16 (1.6%) had unilateral DPOAE screening failure, and 22 (2.2%) had bilateral DPOAE screening failure. A total of 199 (19.6%) babies were found to have at least 1 mutated allele on the NGS for deafness, 11 (1.1%) of whom were homozygous for *GJB2* p.V37I, 6 (0.6%) compound heterozygous for *GJB2* p.V37I and c.235delC, and 1 (0.1%) homoplasmic for m.1555A>G, who may potentially have hearing loss. Among them, 3 babies, 5 babies, and 1 baby, respectively, passed the NHS at birth. Comprehensive audiological assessments in the 9 babies at 3 months identified 1 with slight hearing loss and 2 with mild hearing loss. NGS for common deafness-associated mutations may identify infants with slight/mild or potentially progressive hearing impairment, thus compensating for the inherent limitations of the conventional UNHS.

## Introduction

Hearing loss is one of the most common congenital disorders, with approximately 1 in 1000 newborns affected by bilateral moderate, severe or profound (i.e. >40dBHL) permanent congenital hearing loss (PCHL) [1], [2]. If the criterion of hearing loss is lowered to 15 dBHL, 0.88% of the school-aged population have slight or mild bilateral sensorineural hearing impairment (SNHI) [3]. There is solid evidence that moderate (or more severe) hearing impairment exerts a negative impact on speech, language, and cognitive development [2], and early identification and management may be of great benefit to these children, through improved language, communication, mental health, and employment prospects [4]. Although several risk factors (such as prolonged NICU admission and congenital infections) are associated with PCHL, about 50% of infants with PCHL do not have any known risk factors [5], [6], mandating the implementation of universal newborn hearing screening (UNHS) for both newborns with and without risk factors. The feasibility, cost-efficiency, and benefits of UNHS were supported by several studies [1], [7], [8], [9]. However, UNHS may suffer from 3 inherent limitations. First, since the target condition for the majority of UNHS programs is permanent hearing loss >35 dBHL, children with slight or mild hearing loss will be missed [10]. Second, children with late-onset or progressive hearing loss may not be identified by UNHS, because their hearing is normal or near-normal at birth. Third, even in countries where UNHS has been implemented, it is difficult to approach and screen specific subgroups of infants, such as those born outside of hospitals [11].

With recent advancement in the molecular genetics of hearing impairment, it has been demonstrated that more than 50% of children with SNHI have attributable genetic factors [12], making genetic testing a powerful tool for addressing hearing-impaired children. Among a plethora of deafness genes discovered in the past decade (The Hereditary Hearing Loss Homepage, http://hereditaryhearingloss.org/), mutations in certain genes, such as *GJB2* (or *Cx26*) (MIM *121011), *SLC26A4* (or *PDS*) (MIM *605646), and the mitochondrial 12S rRNA gene (or *MTRNR1*) (MIM *561000) have been shown to be much more prevalent than other genes [13]. Some common *GJB2* mutations, such as p.M34T, p.V37I, and p.L90P are associated with mild-to-moderate SNHI [14]. *SLC26A4* mutations contribute to Pendred syndrome (PS, MIM #274600) or non-syndromic hearing loss (DFNB4, MIM #600791), and some affected patients have progressive or fluctuating hearing loss [15], [16], [17]. Patients with the most common mitochondrial 12S rRNA mutation, m.1555A>G, also demonstrate great variation in the severity of hearing loss progression with age [18]. In other words, some common deafness-associated mutations are associated with mild and/or progressive hearing loss. Accordingly, in this study, we hypothesize that the application of newborn genetic screening for common deafness-associated mutations may compensate for the inherent limitations of UNHS.

## Methods

### Recruitment and study design

From 2009 to 2010, 1017 consecutive newborns in the National Taiwan University Hospital were enrolled in the study. All newborns were subjected to both newborn hearing screening (NHS) and newborn genetic screening (NGS) for deafness. The results of the NGS were then correlated to the results of the NHS. Written informed consent for participation in the project was obtained from the parents of all infants, and all procedures were approved by the Research Ethics Committee of the National Taiwan University Hospital.

### Newborn hearing screening

The babies received a two-step hearing screening using distortion-product otoacoustic emissions (DPOAEs) recorded with a GSI 60 DPOAE system (Grason-Stadler Inc., Milford, NH, USA). The first step of the screening was performed at 48 hours after birth to prevent debris and vernix in the external ear canal from interfering with the DPOAE measurement [19]. Babies who failed to pass the initial screening, either for one or for both ears, were given a second chance to repeat the DPOAE screening before they were discharged from the hospital. Those failing to pass the second step of the hearing screening, either for one or for both ears, were referred to the outpatient clinic for further assessment. The referral rate of the NHS was determined by the proportion of infants who failed both steps of the DPOAE screening.

### Newborn genetic screening for deafness

A bloodspot was obtained within 1 hour after birth. Sample of blood from the heel stick from each infant was spotted onto QIAcard FTA One Spot (Qiagen). For newborn screening, three 3-mm-diameter bloodspots from each blood spot on FTA paper were punched out and used. All DNA samples were extracted using the Chemagic DNA Blood Kit (Chemagen), according to the manufacturer's instructions.

Using our previously generated epidemiologic database [20], the four most common deafness-associated mutations in the Taiwanese population, p.V37I (c.109G>A) and c.235delC of *GJB2*, c.919-2A>G of *SLC26A4*, and mitochondrial m.1555A>G, were included in the NGS panel. In terms of the allele frequency in the hearing-impaired population, these 4 mutations amount to >80% of the known deafness-associated mutations in Taiwanese individuals [20], [21].

**Figure 1:**
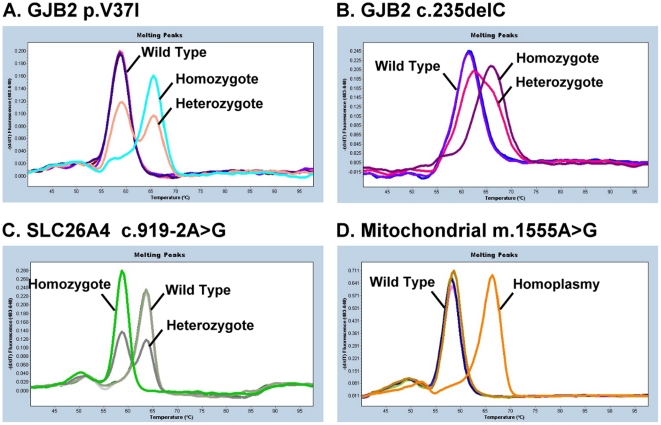
Melting curve analysis of real-time hybridization with FRET probes for the four most common deafness-associated mutations in the Taiwanese population. A, p.V37I of the *GJB2* gene; B, c.235delC of the *GJB2* gene; C, c.919-2A>G of the *SLC26A4* gene; and D, mitochondrial m.1555A>G.

The four mutations were screened using the real-time polymerase chain reaction (PCR) assay with fluorescence resonance energy transfer (FRET) hybridization probes in a LightCycler 480 instrument (Roche) (Fig. 1). The real-time PCR was performed using two primers and two adjacent fluorescent hybridization probes. One probe was labeled with fluorescein at the 3′-end as the donor, and the other probe (acceptor) was labeled with LightCycler (LC) Red fluorophore at the 5′-end, which was phosphorylated at the 3′-end. Real-time PCR was performed in a total volume of 10 µL containing 1 µL of DNA, 3 mM of MgCl_2_, 0.25 µM of each primer, 0.25 of µM fluorescein probe, 0.25 µM of LC Red fluorophore probe, and 1× LightCycler FastStart DNA Master Hybridization Probes Mix (Roche), as provided by the manufacturer. The cycling conditions for real-time PCR in LiC480 were as follows: 95°C for 10 min followed by 50 cycles of denaturation at 95°C for 10 s with a temperature transition rate of 4.4°C/s, annealing at 56°C for 10 s with a temperature transition rate of 2.2°C/s, and extension at 72°C for 10 s, with a temperature transition rate of 4.4°C/s.

### Audiological assessments after discharge

For infants who failed the NHS, another DPOAE test was performed within 1 month after discharge, and those failing to pass the test again were referred to a pediatric otologist for comprehensive audiological assessments at 3 months. The comprehensive audiological assessments included a behavioral observation audiometry (BOA) in a sound field using warble tones, narrow band noise, and live voice; DPOAE testing; and a diagnostic auditory brainstem response (ABR, Nicolet, Bravo, Madison, WI, USA) under sedation to determine the hearing thresholds at 0.5, 1, 2, and 4 kHz [22]. The hearing level of the better ear calculated by four-tone average (0.5, 1, 2, and 4 kHz) was labeled as slight (16∼25 dBHL), mild (26∼40 dBHL), moderate (41∼70 dBHL), severe (71∼95 dBHL), or profound (>95 dBHL) (GENDEAF: http://audiology.unife.it/www.gendeaf.org/index.html) [3].

For infants who passed the NHS but segregated for 2 mutated *GJB2* alleles (i.e., with the p.V37I/p.V37I, p.V37I/c.235delC, and c.235delC/c.235delC genotypes), 2 mutated *SLC26A4* alleles (i.e., with the c.919-2A>G/c.919-2A>G genotype), or the m.1555A>G mutation (i.e., homoplasmic or heteroplasmic), comprehensive audiological assessments including BOA, DPOAE, and ABR were also performed at 3 months to confirm their audiological phenotypes.

### Genetic examination in hearing impaired infants and heterozygous infants

For hearing-impaired infants who revealed an inconclusive genotype in NGS (i.e., segregating 0 or only 1 mutated *GJB2* or *SLC26A4* allele), mutation screening of both exons of *GJB2*, all of the 21 exons of *SLC26A4*, and the whole mitochondrial 12S rRNA gene was completed by direct sequencing [20]. For infants who passed the NHS but carried heterozygous *GJB2* mutations, direct sequencing of the coding region of *GJB2* was also performed.

## Results

### Results of newborn hearing screening

Results of the two-step NHS in the 1017 subjects are summarized in Table 1. Among the 1017 babies, 979 (96.3%) were screened as normal when they bilaterally passed both steps of the DPOAE testing. Of the remaining 38 babies, 16 (1.6%) had unilateral DPOAE screening failure, and 22 (2.2%) had bilateral DPOAE screening failure. The referral rate of the two-step NHS was 3.7%.

**Table 1:** Newborn hearing screening in the 1017 subjects.

OAE results	No. of subjects (%)
Bilateral pass	979 (96.3)
Unilateral failure	16 (1.6)
Bilateral failure	22 (2.2)
Total	1017 (100)
Referral rate	38 (3.7)

### Results of newborn genetic screening for deafness

Of the 1017 subjects, a total of 199 (19.6%) were screened as having at least 1 mutated allele according to the NGS for deafness. Table 2 summarizes their genotypes. As for the *GJB2* mutations, 17 subjects (1.7%) had two mutated alleles, 11 (1.1%) of whom were homozygous for p.V37I and 6 (0.6%) compound heterozygous for p.V37I and c.235delC. None (0%) were homozygous for c.235delC. Nineteen subjects (1.9%) were shown to have 1 c.235delC allele, and 156 subjects (15.3%) were heterozygous for p.V37I. The high carrier rate of *GJB2* p.V37I in this newborn cohort was compatible to that observed in a normal control population in our previous report [23]. In relation to the *SLC26A4* c.919-2A>G mutation, 6 subjects (0.6%) were heterozygous for c.919-2A>G and none (0%) were homozygotes. One subject (0.1%) was found to have the m.1555A>G mutation, with the mutation load detected as “homoplasmy” in hybridization probe testing, which was later confirmed using a restriction enzyme digestion method [24]. None of the 1017 babies segregated for mutations in 2 different genes for deafness, according to NGS.

**Table 2:** Newborn genetic screening in the 1017 subjects.

Genotype	No. of subjects (%)
*GJB2*	
p.V37I/p.V37I	11 (1.1)
p.V37I/c.235delC	6 (0.6)
c.235delC/c.235delC	0 (0)
p.V37I/wt	156 (15.3)
c.235delC/wt	19 (1.9)
*SLC26A4*	
c.919-2A>G/c.919-2A>G	0 (0)
c.919-2A>G/wt	6 (0.6)
Mitochondrial 12S rRNA	
m.1555A>G	1 (0.1)
No mutation identified	818 (80.4)

wt, wild type.

### Analysis of NHS results according to genotypes in NGS

The results of NHS in subjects with different genotypes in NGS are shown in Table 3. Among the 11 babies homozygous for the *GJB2* p.V37I mutation and the 6 babies compound heterozygous for *GJB2* p.V37I and c.235delC, 8 (73%) and 1 (17%), respectively, failed to pass the NHS. In other words, 3 babies homozygous for p.V37I and 5 babies compound heterozygous for p.V37I and c.235delC, with potential hearing problems, were not detected by NHS, indicating that NGS could identify an additional subgroup of affected newborns outside the target area of NHS.

**Table 3:** Newborn hearing screening results in subjects with different genotypes.

		No. of subjects		
Genotype	Total (n)	NHS pass (n)	NHS failure (n)	Failure rate (%)
*GJB2*				
p.V37I/p.V37I	11	3	8	73
p.V37I/c.235delC	6	5	1	17
p.V37I/wt	156	148	8	5
c.235delC/wt	19	18	1	5
*SLC26A4*				
c.919-2A>G/wt	6	6	0	0
Mitochondrial 12S rRNA				
m.1555A>G	1	1	0	0

NHS, newborn hearing screening; wt, wild type.

The failure rates of NHS in the 156 *GJB2* p.V37I heterozygotes and 19 c.235delC heterozygotes were 5% (n = 8) and 5% (n = 1), respectively. Both exons of *GJB2* were then sequenced in the 9 heterozygous babies who failed to pass NHS, yet we did not identify any mutation in the second *GJB2* allele. Likewise, a second *GJB2* mutation was not detected in the other 166 heterozygous babies who passed NHS, indicating that they were probably coincidental carriers of the mutations.

None (0%) of the 6 babies heterozygous for *SLC26A4* c.919-2A>G failed to pass NHS. Probably these babies were only carriers. However, the possibility of Pendred syndrome or non-syndromic DFNB4 could not be completely excluded, because PS or DFNB4 patients might be born with normal hearing or minimal hearing loss, with their hearing deteriorating later during childhood or adolescence [25]. The only subject who was homoplasmic for the mitochondrial m.1555A>G mutation passed the NHS, implying that the hearing of this baby at birth might still be normal or near normal and could not be detected by NHS.

### Audiological assessments after discharge

Among the 38 infants who failed the NHS, 9 (24%) segregated for 2 mutated *GJB2* alleles (including 8 p.V37I homozygotes and 1 p.V37I/c.235delC compound heterozygote), 9 (24%) segregated for 1 mutated *GJB2* allele (including 8 p.V37I heterozygotes and 1 c.235delC heterozygote), and 20 (53%) segregated for none of the 4 deafness-associated mutations (Table 4). Six of the 9 babies with 2 mutated *GJB2* alleles revealed bilateral hearing loss at 3 months, including 2 with moderate hearing loss, 3 with mild hearing loss and 1 with slight hearing loss. The other 3 babies with 2 mutated *GJB2* alleles revealed normal hearing at 3 months. Nevertheless, a close observation of hearing is still warranted in these 3 babies because progressive hearing loss is a common feature in patients with p.V37I [26]. One of the 9 babies with 1 mutated *GJB2* allele demonstrated bilateral moderate hearing loss at 3 months, and 1 of the 20 babies with no mutation detected by NGS showed unilateral profound hearing loss.

**Table 4:** Audiological results (at 3 m) in infants failing NHS.

Genotype	Total (n)	Loss F/U (n)	Normal hearing (n)	Unilateral (n)	Bilateral (n)
2 mutated *GJB2* alleles	9	0	3	0	6^a^
1 mutated *GJB2* allele	9	1	7	0	1^b^
No mutation detected	20	3	16	1^c^	0

a Including 2 babies with moderate hearing loss, 3 with mild hearing loss and 1 with slight hearing loss.

b The baby revealed bilateral moderate hearing loss.

c The baby revealed unilateral profound hearing loss.

Comprehensive audiological assessments were also completed in the 9 infants who passed the NHS but segregated for 2 mutated *GJB2* alleles or homoplasmic m.1555A>G mutation (Table 5). One baby (NGS0071) showed slight hearing loss at 3 months, whereas 2 babies (NGS0032 and NGS0379) showed mild hearing loss at 3 months.

**Table 5:** Audiological results (at 3 m) in infants passing NHS but segregating for an abnormal genotype.

						ABR	threshold	(dBHL)		
Subject no.	Sex	Genotype	Laterality	DPOAE results	0.5 k Hz	1 k Hz	2 k Hz	4 k Hz	average	Audiometry shape
NGS0032	M	*GJB2* p.V37I/c.235delC	bil. symmetric	bil. pass	25	30	30	30	28.8	flat
NGS0071	F	*GJB2* p.V37I/c.235delC	bil. symmetric	bil. pass	15	15	15	20	16.3	flat
NGS0072	F	*GJB2* p.V37I/p.V37I	bil. symmetric	bil. pass	15	10	15	15	13.8	flat
NGS0379	F	*GJB2* p.V37I/c.235delC	bil. symmetric	bil. pass	30	35	35	35	33.8	flat
NGS0586	F	m.1555A>G	bil. symmetric	bil. pass	15	10	10	15	12.5	flat
NGS0598	F	*GJB2* p.V37I/c.235delC	bil. symmetric	bil. pass	10	10	10	15	11.3	flat
NGS0736	M	*GJB2* p.V37I/p.V37I	bil. symmetric	bil. pass	10	5	5	10	7.5	flat
NGS0830	F	*GJB2* p.V37I/c.235delC	bil. symmetric	bil. pass	10	10	10	10	10	flat
NGS0961	M	*GJB2* p.V37I/p.V37I	bil. symmetric	bil. pass	10	10	5	10	8.75	flat

**Figure 2:**
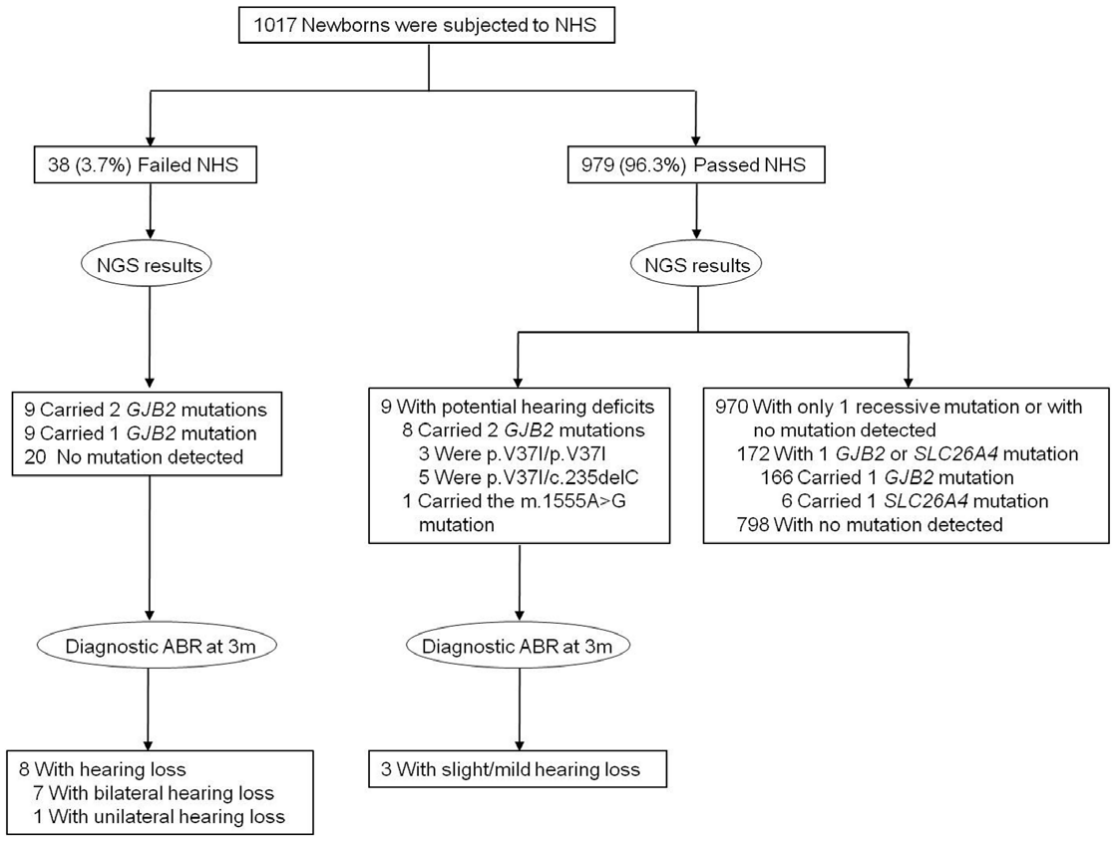
From newborn hearing screening (NHS) and newborn genetic screening (NGS) to identification of hearing loss. Among the 979 newborns who passed NHS, 9 were identified to have potential hearing deficits by NGS. Of these 9, 3 were identified with slight/mild hearing loss at 3 months with diagnostic ABR. ABR, auditory brainstem response.

The clinical utility of NGS was summarized in the flow from NHS and NGS to identification of hearing loss (Fig. 2). Among the 979 newborns who passed NHS, 9 were identified to have potential hearing deficits by NGS. Of these 9, 3 were identified with slight/mild hearing loss at 3 months with diagnostic ABR. In other words, NGS might be useful for identifying slight/mild hearing loss that was not detected by conventional NHS.

### Genetic examination in hearing impaired infants

As shown in Table 4, 1 baby with bilateral moderate hearing loss harbored only a mutated *GJB2* allele, and 1 baby with unilateral profound hearing loss had none of the 4 deafness-associated mutations. Mutation screening of both exons of *GJB2*, all 21 exons of *SLC26A4*, and the whole mitochondrial 12S rRNA gene, was performed in the 2 babies, yet no mutation was identified.

## Discussion

In the present study, we developed a high throughput genetic screening tool to screen 4 common deafness-associated mutations in the Taiwanese population, and applied it to a prospective cohort of 1017 newborns. Our preliminary results revealed that NGS for deafness might compensate for the inherent limitations of conventional UNHS, including failure to identify infants with slight or mild hearing loss, as well as failure to identify infants who may potentially have late-onset or progressive hearing loss during childhood or adolescence.

There have been disputes with regard to whether the goal of UNHS should be to find out infants with slight or mild hearing loss [10], [27]. Several studies demonstrated that slight/mild bilateral SNHI might also have a negative impact on academic performance [28], attention capacity [29], and language skills [30] of children. In a study arguing that slight/mild bilateral SNHI might not affect language, reading, behavior, or health-related quality of life, phonological short-term memory and phonological discrimination were reported to be poorer in children with slight/mild bilateral SNHI [3]. However, including slight/mild losses within the target population of UNHS indicates that the screening program should be altered to achieve higher sensitivity, which inevitably will reduce the specificity of UNHS and greatly increase referral and false-positive rates [27]. To circumvent this difficulty, efforts have been made to ascertain the risk factors for slight/mild SNHI, disclosing that conventional risk factors for neonatal hearing loss, such as craniofacial anomaly, family history of childhood hearing loss and perinatal illness, were not strongly predictive of slight/mild SNHI [31]. On the other hand, the present study provides evidence that NGS, by detecting subjects with genetic mutations associated with mild-to-moderate SNHI, might help in identifying slight/mild SNHI not targeted by conventional UNHS. Although there is still no unanimous consensus concerning the standard management of infants with slight/mild SNHI, referral to early intervention, speech and language monitoring, and provision of resources to parents are recommended [32], [33]. Infants diagnosed as having slight/mild SNHI in the present cohort were referred to rehabilitation facilities for further consultation with an audiologist and a speech pathologist. Even if parents chose not to have their baby fit with a hearing aid immediately, they were provided with information about how to optimize the listening environment for their baby.

To identify infants and children with late-onset or progressive hearing loss at the earliest possible time is also considered important and has been included in the national Early Hearing Detection and Intervention (EHDI) goals (http://www.cdc.gov/ncbddd/ehdi/nationalgoals.htm). However, there is still no efficient way to achieve this goal [11]. In a recent report, progressive hearing impairment was noted in 7 (39%) of the 18 patients who were either homozygous for p.V37I mutation or segregated for a p.V37I allele in compound heterozygosity with a truncating allele, indicating that progression is a common feature for p.V37I [26]. In a Japanese cohort, it was also reported that p.V37I was found mainly in patients diagnosed at a higher age [34]. Progressive hearing loss has also been reported among patients with other *GJB2* mutations, such as p.M34T [35], [36]. These lines of evidence highlight the clinical utility of NGS for certain *GJB2* mutations in identifying infants who are likely to have late-onset or progressive hearing loss, necessitating a close observation of hearing in these affected individuals. The potential benefits of identifying subjects with *SLC26A4* mutations or the mitochondrial m.1555A>G mutation might even be greater. For subjects with *SLC26A4*-mutation-associated Pendred syndrome or non-syndromic DFNB4, attacks of acute sensorineural hearing loss could be prevented by avoiding head trauma or abrupt barometric pressure changes [25], thus halting the progression or fluctuation of hearing impairment. The severity of hearing loss due to m.1555A>G mutation is modulated by several factors [24]. Especially, the use of aminoglycosides should be avoided in individuals with the mutation, because m.1555A>G is well established as a predisposing factor for aminoglycoside ototoxicity [37].

Interestingly, a recent study reported 32 of 108 cochlear implant (CI) recipients (29.6%) born in Illinois during or after 2003 passed UNHS, demonstrating the limitation of the current UNHS in early identification of delayed-onset SNHI [38]. The authors also pointed out that this problem could not be solved by recent JCIH guidelines, which suggest reevaluation by 24 to 30 months of age in children with known risk factors who pass UNHS [38]. Repeated hearing screening in all children provides a solution, but it might be rather costly as well as unfeasible once the babies are discharged from hospitals or clinics. On the other hand, NGS might be helpful in identifying a group of children with increased risk in developing SNHI for whom a close audiological assessment should be implemented. In the Illinois series, a substantial proportion of CI recipients would have been identified by NGS, since among the 32 CI recipients who passed UNHS, 6 were diagnosed as having connexin mutations, and 7 were found to have cochlear malformations, which might be associated with *SLC26A4* mutations.

With regard to the difficulty in approaching and screening specific subgroups of infants such as those born outside of hospitals, NGS might also serve as an alternative solution. Obtaining a few drops of blood from a heel stick within the first 2 or 3 days of life is a minimally invasive procedure, and can be conducted by a nurse or a midwife with basic training. In addition, the new generation of DNA cards, such as filter blotters, Guthrie cards, and FTA cards, are easy to collect, transport, and store.

Offering parents reproductive choices (prenatal diagnosis) has been considered a benefit of expanded newborn screening programs [39]. Although this might be true for certain rare diseases, its clinical implications in deafness should be scrutinized with caution. Some deaf advocates argue that deafness is not a disability, and are against screening for hearing defects [40]. Even for hearing parents of deaf children, it has been documented that few of them would use genetic information to terminate an affected pregnancy, although most recorded a positive attitude toward prenatal genetic testing for deafness [41], [42].

New ethical questions might emerge with the institution of NGS for deafness, including risks of discrimination or stigmatization, respect for autonomy of persons to make their own decisions, and parental anxiety resulting from a false positive test or the carrier status of a recessive mutation, as other newborn screening panels [39]. For hereditary hearing impairment, of which the majority of cases are inherited in an autosomal recessive manner, identification of healthy carriers could be of special concern because it might lead to unjustified parental anxiety about the health of their baby [43]. In the present study, 175 babies were found to carry 1 *GJB2* mutation, and 6 babies were found to carry 1 *SLC26A4* mutation; among them, 9 babies and no baby failed the NHS at birth, respectively. These 9 babies were managed as other newborns who failed the NHS according to the national guidelines, i.e., another DPOAE test within 1 month after discharge, followed by comprehensive audiological assessments at 3 months if indicated. Seven of the 9 babies had normal hearing at 3 months (Table 4), and were regarded as coincidental carriers, given the high frequency of the *GJB2* p.V37I allele in the Taiwanese population and the absence of a second *GJB2* mutation in these babies. One baby revealed bilateral moderate hearing loss at 3 months, and genetic counseling was performed as hearing-impaired patients with only 1 *GJB2* mutated allele detected [44]. By contrast, for carriers who passed the NHS, no further audiological studies were performed. The great majority of these babies were p.V37I heterozygotes, and were regarded as coincidental carriers given the high frequency of the *GJB2* p.V37I allele in the Taiwanese population. The parents were assured that their babies were not at an increased risk of developing hearing impairment, and access to genetic counseling was provided whenever necessary to minimize potential stress for families.

Of special note, deafness-associated mutations should be adjusted if NGS for deafness is to be implemented in other populations because different ethnic groups show different mutation spectra for each deafness gene. Although mutations of the connexin 26-encoding *GJB2* gene are the most common cause of hereditary hearing loss in most world populations, they occur at different frequencies across populations. To screen *GJB2* mutations in Caucasians, c.35delG, p.M34T, and p.L90P should be included in the screening panel instead of p.V37I and c.235delC, since these mutations are more common in Caucasians [14]. Likewise, to screen *GJB2* mutations in Ashkenazi Jews, c.167delT should be included [45]. Leading to Pendred syndrome, the most common form of syndromic deafness [46], as well as to DFNB4, a common form of non-syndromic deafness with enlarged vestibular aqueduct (EVA), mutations of *SLC26A4* might be the second most frequent cause of hereditary hearing loss worldwide. Common *SLC26A4* mutations also differ across populations. To screen *SLC26A4* mutations, p.T416P and c.1001+1G>A should be covered in populations of northern European ethnicity [47], [48], [49], [50], whereas p.H723R should be included if screening is to be performed in Japanese [51] or Koreans [52]. The m.1555A>G mutation appears to be a common cause of hearing impairment worldwide, and has been reported in 2–5% of sensorineural hearing-impaired Caucasians [53], [54], Japanese [55], and Southeast Asians [56]. In a European birth cohort unselected for hearing loss, the prevalence of m.1555A>G was as high as 1 in 520 [57], attesting to the necessity for conducting national genetic screening prior to aminoglycoside administration. For Spanish subjects, the p.Q829X mutation of the *OTOF* gene (MIM *603861) should be added due to its high prevalence in patients with prelingual SNHI [58].

Some limitations of the present study deserve discussion. First, only common, known deafness-associated mutations are included in the current screening panel. This might lead to a false assurance in individuals with rare or novel mutations because of normal NGS results. This limitation, however, might be overcome with the advent of the next-generation sequencing technology, as massively parallel sequencing has recently been proven as an effective tool for addressing hereditary hearing loss [59]. Second, as a preliminary report, the present study documented the audiological results in 9 infants passing NHS, but segregating for an abnormal genotype, until 3 months. Although 3 of them were confirmed as having slight/mild SNHI, the follow-up period of 3 months is apparently too short to elucidate the frequency of late-onset hearing loss. Long-term follow-up of the present cohort is thus necessary to delineate correlations between NHS and NGS findings. Third, genetic heterogeneity of hereditary hearing loss might preclude a precise interpretation of NGS results. For instance, genetic diagnosis is complicated by the fact that 10%–50% of affected subjects with *GJB2* mutations carry only 1 mutant allele. Although the del(*GJB6-D13S1830*) mutation, through eliminating a *cis*-regulatory element of *GJB2*, provided an explanation for the deafness in as many as 30%–70% of affected *GJB2* heterozygotes in some populations [60], [61], the etiology remained unclear for the others. These affected heterozygotes pose a diagnostic dilemma: their hearing loss might be attributed to an unrecognized *GJB2* mutation, or they might be merely coincidental carriers with hearing loss unrelated to *GJB2*. Similarly, it is also difficult to interpret the genetic results in babies who passed NHS and segregated only 1 mutated *GJB2* allele on NGS, although it can be inferred that a greater proportion of these babies are coincidental carriers as compared to “affected” heterozygotes. Nonetheless, NGS for deafness is still useful in identifying babies with 2 mutated *GJB2* alleles, for whom the genetic diagnosis is more straightforward. These babies require a close audiological observation or intervention because their genotypes are associated with mild-to-moderate, progressive, or late-onset hearing impairment.

In a retrospective report, it was demonstrated that some patients with 2 *GJB2* mutations could not be identified with UNHS [62]. By contrast, the present study might be the first prospective study to have conducted hearing screening in conjunction with genetic screening in all newborns and longitudinal follow-up of these infants. For babies with a definite genetic diagnosis on NGS (i.e., with 2 mutated *GJB2* alleles, 2 mutated *SLC264* alleles, homoplasmic or heteroplasmic m.1555A>G mutation) who reveal normal hearing at 3 months, comprehensive audiological evaluation will be repeated at 1 year, whereas for normal hearing babies with 1 mutated *GJB2* or *SLC264* allele, adequate genetic counseling will be implemented, and additional genetic study (such as sequencing of the whole gene) will be provided whenever necessary. Once hearing impairment develops in these babies, the management protocol will be switched to the conventional management protocol for pediatric SNHI regardless of their genotypes. Long-term follow-up of this cohort might provide insight into how and to what extent genetic mutations exert their influence on the development of pediatric hearing impairment, as well as provide important audiological information about the natural history of specific genetic mutations, such as the *GJB2* p.V37I mutation.

**Figure 3:**
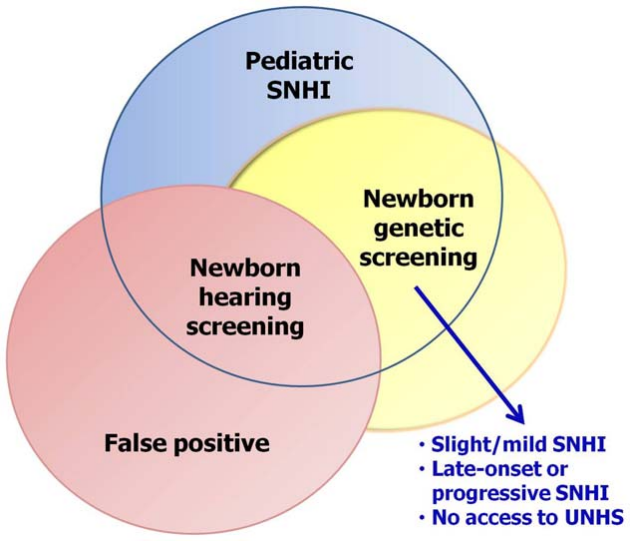
Clinical utility of newborn genetic screening (NGS) for deafness. NGS for common deafness-associated mutations, by detecting subjects with mutations associated with mild-to-moderate, progressive, or late-onset hearing impairment, may compensate for the inherent limitations of conventional universal newborn hearing screening (UNHS), including failure to identify infants with slight or mild hearing loss, as well as failure to identify infants who potentially have late-onset or progressive hearing loss during their childhood or adolescence. For infants born outside of hospitals and who do not have an access to UNHS at birth, NGS may also serve as an alternative. SNHI, sensorineural hearing impairment.

In conclusion, the present study revealed that NGS for common deafness-associated mutations, by detecting subjects with mutations associated with mild-to-moderate, progressive, or late-onset hearing impairment, might compensate for the inherent limitations of conventional UNHS (Fig. 3). For infants born outside of hospitals and who do not have access to UNHS at birth, NGS might also serve as an alternative. The benefits of NGS for deafness would be maximized with the construction of a well-designed infrastructure to support testing, counseling, education, treatment, and follow-up. Despite its clinical utility, the authors would like to emphasize that the role of NGS for deafness is to augment the armamentarium of UNHS instead of replacing UNHS, given that a genetic cause could not be identified in many hearing-impaired children. To our knowledge, this pilot report might be among the first to demonstrate the clinical utility of NGS for deafness. A nation-wide screening is currently underway to confirm the long-term benefits of NGS for the detection of deafness.
